# A Web-Based Service Delivery Model for Communication Training After Brain Injury: Protocol for a Mixed Methods, Prospective, Hybrid Type 2 Implementation-Effectiveness Study

**DOI:** 10.2196/31995

**Published:** 2021-12-09

**Authors:** Melissa Miao, Emma Power, Rachael Rietdijk, Melissa Brunner, Deborah Debono, Leanne Togher

**Affiliations:** 1 University of Technology Sydney Sydney Australia; 2 University of Sydney Sydney Australia

**Keywords:** implementation science, patient-outcome assessment, internet interventions, acquired brain injury, delivery of health care, caregivers, speech-language pathology

## Abstract

**Background:**

Acquired brain injuries (ABIs) commonly cause cognitive-communication disorders, which can have a pervasive psychosocial impact on a person’s life. More than 135 million people worldwide currently live with ABI, and this large and growing burden is increasingly surpassing global rehabilitation service capacity. A web-based service delivery model may offer a scalable solution. The Social Brain Toolkit is an evidence-based suite of 3 web-based communication training interventions for people with ABI and their communication partners. Successful real-world delivery of web-based interventions such as the Social Brain Toolkit requires investigation of intervention implementation in addition to efficacy and effectiveness.

**Objective:**

The aim of this study is to investigate the implementation and effectiveness of the Social Brain Toolkit as a web-based service delivery model.

**Methods:**

This is a mixed methods, prospective, hybrid type 2 implementation-effectiveness study, theoretically underpinned by the Nonadoption, Abandonment, Scale-up, Spread, and Sustainability (NASSS) framework of digital health implementation. We will document implementation strategies preemptively deployed to support the launch of the Social Brain Toolkit interventions, as well as implementation strategies identified by end users through formative evaluation of the Social Brain Toolkit. We will prospectively observe implementation outcomes, selected on the basis of the NASSS framework, through quantitative web analytics of intervention use, qualitative and quantitative pre- and postintervention survey data from all users within a specified sample frame, and qualitative interviews with a subset of users of each intervention. Qualitative implementation data will be deductively analyzed against the NASSS framework. Quantitative implementation data will be analyzed descriptively. We will obtain effectiveness outcomes through web-based knowledge tests, custom user questionnaires, and formal clinical tools. Quantitative effectiveness outcomes will be analyzed through descriptive statistics and the Reliable Change Index, with repeated analysis of variance (pretraining, posttraining, and follow-up), to determine whether there is any significant improvement within this participant sample.

**Results:**

Data collection commenced on July 2, 2021, and is expected to conclude on June 1, 2022, after a 6-month sample frame of analytics for each Social Brain Toolkit intervention. Data analysis will occur concurrently with data collection until mid-2022, with results expected for publication late 2022 and early 2023.

**Conclusions:**

End-user evaluation of the Social Brain Toolkit’s implementation can guide intervention development and implementation to reach and meet community needs in a feasible, scalable, sustainable, and acceptable manner. End user feedback will be directly incorporated and addressed wherever possible in the next version of the Social Brain Toolkit. Learnings from these findings will benefit the implementation of this and future web-based psychosocial interventions for people with ABI and other populations.

**Trial Registration:**

Australia and New Zealand Clinical Trials Registry ACTRN12621001170819; https://anzctr.org.au/Trial/Registration/TrialReview.aspx?ACTRN=12621001170819, Australia and New Zealand Clinical Trials Registry ACTRN12621001177842; https://anzctr.org.au/Trial/Registration/TrialReview.aspx?ACTRN=12621001177842, Australia and New Zealand Clinical Trials Registry ACTRN12621001180808; https://anzctr.org.au/Trial/Registration/TrialReview.aspx?ACTRN=12621001180808

**International Registered Report Identifier (IRRID):**

DERR1-10.2196/31995

## Introduction

### Background

More than 135 million people worldwide currently live with acquired brain injuries (ABIs), including traumatic brain injury and stroke [1]. ABIs commonly cause cognitive-communication disorders in which underlying problems with working memory, organization, executive function, self-regulation, or a combination of these, affect the communication skills needed for everyday exchanges such as conversations, explanations, and stories [2]. Cognitive-communication disorders can thus have a pervasive impact on a person’s social participation and relationships [3], employment [4,5], and mental health [6], while presenting concurrent health, psychosocial, and economic challenges for their families and communities [7-9].

The growing psychosocial burden of ABI increasingly surpasses the global rehabilitation service capacity to address it [1], including national-scale shortages in public speech-language pathology services to manage communication difficulty [10]. Inversely, families of people with ABI, particularly from rural and remote areas, experience logistical and access challenges when seeking face-to-face health care, leading carers to express their need for locally accessible support [11]. The equitable and scalable delivery of communication rehabilitation for people with ABI is therefore a global health service challenge [1], demanding consideration of alternative and complementary service delivery models to meet the psychosocial needs of this population and their communities now and into the future.

In response to these challenges, an evidence-based suite of web-based interventions known as the Social Brain Toolkit [12] is currently in development. The project was cocreated with stakeholders, including people with ABI and their communication partners, clinicians, partnering organizations, and policy makers. These stakeholders have been attending, and are included in, regular steering and advisory committee meetings. They have provided feedback on early prototypes and have been involved in the planning of implementation strategies and now the formative evaluation of the Social Brain Toolkit products. The aim of the Social Brain Toolkit is to provide scalable communication training to people with ABI and their communication partners, including family members, friends, partners, paid support workers, and clinicians. The Social Brain Toolkit comprises 3 web-based interventions: (1) convers-ABI-lity, a conversation skills training program for adults with ABI and familiar communication partners such as family members, partners, and friends; (2) interact-ABI-lity, web-based communication training for unfamiliar communication partners of people with ABI, such as paid support workers and the general public; and (3) social-ABI-lity, social media training for people with ABI seeking to communicate and connect on the web. interact-ABI-lity and social-ABI-lity are self-directed web-based courses, whereas convers-ABI-lity includes self-directed web-based content between telehealth sessions with a speech-language pathologist. The need for and format of these communication training courses were identified together with stakeholders, including people with ABI and their communication partners, with the aim of improving the quality of life and psychosocial outcomes of people with ABI and their communities.

The communication skills of communication partners can have a positive or detrimental effect on the communication skills of people with ABI [13-15]. Therefore, interact-ABI-lity and convers-ABI-lity deliver an evidence-based [16] intervention known as communication partner training (CPT), which involves training communication partners to facilitate the communication [17] of the person with ABI. CPT is recommended in international guidelines as best practice management of cognitive-communication disorders after ABI [18,19], and the convers-ABI-lity intervention delivers the core therapeutic content of the existing efficacious CPT programs TBI Express [20] and TBIconneCT [21,22]. convers-ABI-lity is a conversion and streamlining of the content of these programs into both asynchronous self-directed activities and synchronous telehealth speech-language pathology sessions. interact-ABI-lity delivers CPT as an asynchronous, self-directed web-based educational intervention.

In addition, the Social Brain Toolkit contains the social-ABI-lity intervention, which provides people with ABI with training in communication through social media. This is because people with ABI who use social media for connection are likely to experience difficulties in web-based interactions that are similar to those experienced in real-world interactions [23]. The social-ABI-lity intervention in the Social Brain Toolkit is an educational intervention based on new recommendations to support and train people with ABI in the safe and effective use of social media, with a view to increasing their social participation, enabling recovery of social communication skills, and promoting a sense of self or identity after ABI [24].

Web-based access to psychosocial interventions such as the Social Brain Toolkit are promising not just for the possibility of improving the scalability and accessibility of interventions, but also for their potential to reduce inequities in access to psychosocial support. Even before global shifts to web-based health care during the COVID-19 pandemic [25], people with ABI frequently accessed language and cognitive training on the web, with older patients and rural residents even more digitally engaged than younger and urban users [26]. When delivered on a national scale, equivalent web-based service delivery models in mental health have demonstrated the ability to overcome entrenched health care access barriers such as socioeconomic and indigenous status, and to enable access to users who otherwise do not access traditional face-to-face care [27,28]. However, web-based service delivery models face numerous implementation challenges. Even clinically effective digital health interventions struggle to be sustained as a long-term service delivery option [29] for reasons beyond their clinical effectiveness, including costs and workflow changes associated with their delivery [30]. Although there is an established and varied evidence base exploring web-based psychosocial interventions [31,32], there is limited implementation science research over and above these clinical trials to determine how these interventions might be successfully implemented and sustained as part of routine clinical care [31]. Therefore, a specific focus on implementation, especially in early research collaboration with end users, has been recommended for future research into web-based psychosocial care [27-31].

Therefore, real-world evaluation of the implementation of the Social Brain Toolkit interventions demands (1) collaborative involvement of end users, (2) a hybrid implementation-effectiveness research design [33] to expedite the incorporation of implementation learnings into intervention design, and (3) a theoretical underpinning in a digital health implementation framework that reflects the complexity of real-world implementation. Thus, this hybrid implementation-effectiveness study will be underpinned by an implementation theory that is both specific to digital health and based on a complexity approach: the Nonadoption, Abandonment, Scale-up, Spread, and Sustainability (NASSS) framework of eHealth implementation [34-36]. This framework will be used to support the more comprehensive identification of real-world complexities in the scale-up, spread, and sustainability of the Social Brain Toolkit.

### Aims

In this study, formative evaluation of the implementation of the Social Brain Toolkit by end users in the community will be used to guide intervention development and implementation [37] to support these interventions to reach and meet community needs in a feasible, scalable, sustainable, and acceptable manner. Therefore, guided by domains of the NASSS framework [34,35], in this hybrid implementation-effectiveness study, we aim to answer the following implementation questions:

1. Who uses these interventions and what are their characteristics (domains 1 and 4)? What implementation strategies can be used to improve intervention reach?
2. In what geographical locations and health care and social contexts are the interventions used (domains 3 and 5-6)? What implementation strategies can be used to improve intervention reach?
3. Do users complete the interventions as intended (domain 4)? Why or why not? What implementation strategies can be used to improve intervention adherence and fidelity?
4. How usable is the technology for those completing the interventions (domain 2)? What changes can be made to improve usability?
5. What barriers, facilitators, and workarounds do users experience when completing these interventions (domains 1-7)? What strategies, facilitators, and workarounds can be used to improve future implementation?
6. How satisfied are users with the interventions (domain 3)? What changes can be made to increase user satisfaction?
7. What is the cost of delivering each web-based intervention, and how does this compare with face-to-face delivery (domain 3)?

We seek to determine intervention effectiveness as follows:

1. Do people who complete the interact-ABI-lity course have improvements in their self-efficacy and knowledge about communicating with people with ABI?
2. Do people who complete the social-ABI-lity course have improvements in their self-efficacy and knowledge about communicating safely and successfully on social media?
3. Do people who complete the convers-ABI-lity program have improved communication and quality-of-life outcomes?

## Methods

### Design

This study uses a prospective hybrid type 2 implementation-effectiveness design [33] and is registered on the Australia and New Zealand Clinical Trials Registry: interact-ABI-lity ACTRN12621001170819 (Universal Trial Number [UTN] U1111-1266-6628); social-ABI-lity ACTRN12621001177842 (UTN U1111-1268-4909); and convers-ABI-lity ACTRN12621001180808 (UTN U1111-1268-4849). The Social Brain Toolkit is well suited to a hybrid implementation-effectiveness design [37] because its interventions present minimal risk [16], with indirect evidence supporting effectiveness [22-24] and strong face validity supporting applicability to a web-based delivery method [21,22]. A prospective hybrid type 2 design especially reflects a collaborative ethos because it allows formative evaluation by end users to inform the refinement and improvement of both the clinical interventions and their implementation processes [37]. Preemptive implementation strategies will be devised during intervention development, including through consultation with people with ABI, communication partners, and clinicians supporting people with ABI, and people with experience implementing digital health [38], as well as reference to current implementation science literature [39]. Additional user-identified implementation strategies will be determined as part of the formative evaluation processes in this study, rather than solely a priori, in keeping with our collaborative ethos and approach. The deployment of additional user-identified strategies will enable us to identify and potentially address persisting and unanticipated implementation barriers or shortcomings of our preemptive implementation strategies.

### Data Collection

To obtain these data, we will use a mixed methods design for both implementation (Multimedia Appendix 1) and effectiveness (Multimedia Appendix 2) [40-42] data collection.

#### Interviews

Intervention usability and user experience and satisfaction will be formatively evaluated through interviews. The first 30 minutes of the 90-minute interview will involve a think-aloud [43] review of the user interface through screen sharing of the web-based platform. The think-aloud method is a robust and flexible research technique to test usability by providing valuable and reliable information of users’ cognitive processes while completing a task [43]. This think-aloud task will be followed by a 60-minute qualitative interview, with interview questions developed using the NASSS framework of digital health implementation [34] to prompt discussion of multiple issues within this time frame (see Multimedia Appendices 3-7 for interview protocols). We will conduct the interviews as soon as possible after a user’s completion of the course to facilitate recollection of the intervention experience. We will conduct the interviews individually and with communication partners, depending on participant preference and availability. Data collection will occur entirely on the web, with interviews conducted through secure videoconferencing on Microsoft Teams (Microsoft Corporation) software [44]. The interview will be audio and video recorded using the built-in recording functions of the videoconferencing platform. Interview recordings will be transcribed verbatim for analysis.

#### Surveys

For all 3 interventions in the Social Brain Toolkit, we will use pre- and postintervention surveys within the intervention platforms to obtain implementation outcomes, including user demographic information, and qualitative and quantitative patient-reported experience measures (Multimedia Appendix 1). We will conduct the surveys completely on the web. The surveys are based on the NASSS framework domains (see Multimedia Appendices 8-10 for survey protocols). We will measure intervention effectiveness using questionnaires that probe patient-reported outcome measures such as self-ratings of confidence in communicating with someone with ABI or using social media (Multimedia Appendix 2).

#### Analytics

We will collect web analytics (Multimedia Appendix 1) for each intervention over a 6-month sampling frame. This will enable user fidelity and adherence to the interventions to be examined.

#### Clinical Outcomes

Clinical outcomes (Multimedia Appendix 2) examining the effectiveness of convers-ABI-lity will be collected in a parallel study. As interact-ABI-lity and social-ABI-lity are educational interventions, their effectiveness will be examined by measuring knowledge through preintervention, postintervention, and follow-up multiple-choice questions.

### Analysis

#### Qualitative

To examine effectiveness, 2 experienced speech-language pathologists will review the lists of strategies generated by users of the social-ABI-lity and interact-ABI-lity courses, and code them as appropriate or inappropriate using a consensus rating procedure (Multimedia Appendix 2). To examine implementation, the first author (MM) will conduct deductive content analysis [45] based on the NASSS framework [34] of both free-text survey responses and interview data (Multimedia Appendix 1). Coding will be managed in NVivo 12 Pro (QSR International Pty Ltd) [46] or Microsoft Excel 2016 [47] (Microsoft Corporation) software.

#### Quantitative

We will prospectively measure implementation outcomes in relation to the following:

1. Preemptive strategies deployed to support the implementation of the Social Brain Toolkit at launch, devised through current implementation evidence [39] and stakeholder input from a separate study [38].
2. Additional user-identified strategies subsequently obtained through formative evaluation of the interventions by end users.

To observe any potential influence of these implementation strategies and factors on implementation success and the time lag of impact, we will record the following:

1. A detailed description of each implementation strategy and its rationale.
2. A detailed timeline of each strategy’s deployment.
3. Effectiveness and implementation outcomes over time.

We will calculate descriptive statistics of implementation measures, including user demographic characteristics, satisfaction ratings, percentage completion, and total number of users (Multimedia Appendix 1). We will tabulate descriptive statistics stratified by time and by whether users complete the interventions. Descriptive statistical analysis will be conducted using RStudio software (RStudio Inc) [48].

Clinical assessment data for conversation skills and quality of life (Multimedia Appendix 2) [40-42] will be analyzed using the Reliable Change Index [49] to determine whether individual participants had any clinically significant changes. Patient-reported outcome measures for interact-ABI-lity and social-ABI-lity, such as self-ratings of confidence, will be analyzed using repeated measures analysis of variance, with 3 levels (pretraining, posttraining, and follow-up) to determine whether there is any significant improvement within this participant sample. These data will also be managed using appropriate statistical software such as Microsoft Excel 2016 (Microsoft Corporation) [47].

Finally, theoretically underpinned by the third domain of the NASSS framework [34] examining the value proposition of an intervention, we will use the web analytics data for each intervention to calculate web-based health care costs and equivalent face-to-face costs using a bottom-up costing approach [50]. With an initial focus on the Australian context from which the Social Brain Toolkit is developed, we will refer to nationally recognized cost guides such as the Australian Medicare Benefits Scheme [51] to obtain relevant unit costs. Costs will be calculated in Australian dollars, with equivalent conversions reported in euros and US dollars, using RStudio software (RStudio Inc) [48].

### Rigor

For qualitative implementation data, a second author (EP, RR, LT, MB, or DD) will verify a random 25% of the total codes from the (1) first interview for interact-ABI-lity, (2) first interview for social-ABI-lity, (3) first clinician interview for convers-ABI-lity, and (4) first interview with a person with ABI and their communication partner for convers-ABI-lity. Any discrepancies will be resolved through research team discussion and consensus before the first author (MM) proceeds to code the remaining interviews. Qualitative effectiveness data will be managed by 2 experienced speech-language pathologists through a consensus rating procedure. For quantitative implementation and effectiveness data, a second author (EP, RR, LT, MB, or DD) will review outputs, and the first author (MM) will consult a biostatistician for support as necessary. For quantitative costing data, analysis will be conducted in consultation with a health economist and the clinical research team, with calculation methods, rationales, and references transparently reported. Overall results will be reported according to the Standards for Reporting Implementation Studies [52].

### Participants

#### Inclusion and Exclusion Criteria for Users of interact-ABI-lity and social-ABI-lity

As social-ABI-lity and interact-ABI-lity are publicly available web-based courses, all users of the courses within the sample frame will be included in data collection and analysis of survey and analytic data, with no restrictions of inclusion and exclusion criteria. A minimum of 5 users of the social-ABI-lity course (ie, people with ABI) and interact-ABI-lity courses (ie, communication partners such as paid support workers, friends, and family members) will be invited to participate in further implementation interviews. This number of users interviewed is consistent with the think-aloud methods described in the study design [43], which are used to refine the usability of the courses. The internationally recognized industry standard [53] is for a minimum of 5 users to undergo a formative usability interview evaluation [53], because only so many users are required to identify up to 90% of the usability issues [54] before there are diminishing returns for the product cycle [53]. However, a maximum variation sample of these interviewees will be sought if and where possible.

If people with ABI completed the social-ABI-lity course with the assistance of a friend, partner, family member, or other individual, this person will also be invited through the course user to be interviewed together or individually, depending on individual preference or availability. Therefore, interview participants must meet the following criteria:

1. They must have registered for, and used, at least some modules of interact-ABI-lity or social-ABI-lity, as verified by course records.
2. They must have indicated consent at course enrollment to be contacted for further research participation opportunities related to the course.
3. They must have provided informed written consent to participate in the interview. For users who have an ABI, capacity to consent will be determined during a video call with a qualified speech-language pathologist according to our adapted consenting process protocol that includes relevant questions adapted from the University of California, San Diego, Brief Assessment of Capacity to Consent instrument [55]. People with ABI without the ability to respond correctly to all 5 questions presented using supported communication strategies, as outlined in Multimedia Appendix 11 [55], will be excluded from the study.
4. They must be aged ≥18 years.
5. They must have adequate English proficiency to participate in the study without the aid of an interpreter, with functional reading skills in English.

There are no restrictions on any other factors (eg, gender, clinical experience, or geographical location) for interview participants who have used interact-ABI-lity and social-ABI-lity. Where possible, variation in these factors is preferred to obtain a purposive, maximum variation sample of user experiences of the interventions.

#### Inclusion and Exclusion Criteria for Users of convers-ABI-lity

Interviews will be conducted with all 10 people with ABI and their 10 communication partners who have completed the pilot version of convers-ABI-lity, as well as the 5 clinicians delivering the intervention. Participants with ABI must meet the following criteria:

1. They must have had a definite moderate-severe ABI at least 6 months previously based on the Mayo Classification Scheme [56] (at least one of the following: loss of consciousness of 30 minutes or more, posttraumatic amnesia lasting ≥24 hours, worst Glasgow Coma Scale total score in the first 24 hours of <13, or evidence of a significant brain imaging abnormality). People with a non–traumatic brain injury (restricted specifically to the etiologies of stroke, hypoxic injury, brain tumor, poisoning, and infection) will also be eligible to participate.
2. They must have been discharged or partially discharged from hospital and be able to spend time at home on a regular basis.
3. They must have significant social communication skills deficits (either self-identified or identified by a usual communication partner).
4. They must have insight into their social communication skills deficits.
5. They must be aged 18-70 years.
6. They must have adequate English proficiency for completing assessment tasks without the aid of an interpreter.
7. They must have functional reading skills in English.
8. They must have a communication partner with whom they interact regularly who is willing to participate in the research interviews and training program.

The exclusion criteria for participants with ABI are as follows:

1. Aphasia of a severity such that it prevents any participation in conversation.
2. Severe amnesia, which would prevent participants from providing informed consent, as evaluated using the University of California, San Diego, Brief Assessment of Capacity to Consent instrument [55].
3. Dysarthria of a severity such that it significantly reduces intelligibility during conversation, as evaluated by the researcher.
4. Drug or alcohol addiction, which would prevent participants from reliably participating in sessions.
5. Active psychosis.
6. Co-occurring degenerative neurological disorder, more than one episode of moderate-severe ABI, or premorbid intellectual disability.

Family members, friends, or paid support workers participating in the study must meet the following criteria:

1. They must regularly interact with a person with ABI (ie, at least once a week). This person with ABI must have had the ABI at least 6 months previously.
2. They must have known the person with ABI for at least 3 months.
3. They must not have sustained a severe ABI themselves.
4. They must be aged ≥18 years.

Speech-language pathologists delivering convers-ABI-lity must meet the following criteria:

1. They must be currently employed in a clinical speech-language pathology role.
2. At least 20% of their caseload must comprise people with ABI.

### Ethics

This research has received ethical approval from the University of Technology Sydney (UTS) Health and Medical Research Ethics Committee (ETH21-6111) to conduct interviews with users of social-ABI-lity and interact-ABI-lity. The UTS Health and Medical Research Ethics Committee has also ratified (ETH21-5899) an approval by the Western Sydney Local Health District Human Research Ethics Committee (2019/ETH13510) to conduct interviews with users of convers-ABI-lity and collect demographic and web analytic data for all 3 interventions.

Users of the interact-ABI-lity and social-ABI-lity courses provide their email addresses at registration and indicate whether they consent to participate in follow-up research related to these courses. Consenting users of the courses will be invited to provide informed written consent to participate in a follow-up interview using an accessible, lay-language participant information and consent form. To ensure informed consent, the form will be adapted with visual supports and explained through video call for people with ABI, outlining the full burden and risks of research participation. Screening for capacity to consent is described in the aforementioned inclusion criteria (Multimedia Appendix 11).

Participants with ABI and their communication partners will be paid for their interviews at the 2021 hourly rate recommended by Health Consumers New South Wales [57]. Reimbursement for people with ABI and their communication partners is viewed as critical to recognizing the value of their lived experience of ABI, caring, and health care. It also aims to minimize any undue burden of research participation. Potential participants will be advised of this arrangement in the participant information form to facilitate decision-making around any potential economic burden of participation.

## Results

### Research Funding

Australian National Health and Medical Research Council Postgraduate Scholarship funding was granted in November 2019, UTS Centre for Social Justice & Inclusion Social Impact funding was granted in April 2021, and icare New South Wales Quality of Life funding was received in November 2019.

### Ethical Approval

Ethical approval was received from the UTS Health and Medical Research Ethics Committee (ETH21-6111) on June 29, 2021, and the Western Sydney Local Health District Human Research Ethics Committee (2019/ETH13510) on June 11, 2021, with ratification by the UTS Health and Medical Research Ethics Committee (ETH21-5899) on June 29, 2021.

### Participant Enrollment

At manuscript submission on July 13, 2021, 85 participants had enrolled in interact-ABI-lity, with 85 completed entry surveys, 6 course completions, and 8 completed exit surveys. No interview data had yet been collected. Data collection for interact-ABI-lity commenced on July 2, 2021, and is expected to conclude on January 2, 2022, after a 6-month sample frame of analytics. By July 13, 2021, 1 participant had been recruited to participate in the convers-ABI-lity study, with no data yet collected. Data collection is expected to commence on July 26, 2021, and conclude on March 26, 2022. By July 13, 2021, no participant had been recruited to participate in the social-ABI-lity study. Data collection is expected to commence on December 1, 2021, and conclude on June 1, 2022, after a 6-month sample frame of analytics.

### Analysis and Findings

Data analysis will occur concurrently with data collection until mid-2022. Results are expected for publication during late 2022 and early 2023.

## Discussion

### A Dual Focus on Implementation and Effectiveness

As the global burden of ABI grows, our communities and health care system must find a scalable, feasible, acceptable, and accessible health care service delivery solution to address the psychosocial burden of this condition [1]. A concerted focus on implementation is essential to ensure the successful and sustained uptake of health interventions, without which even efficacious treatments have failed to be adopted, implemented, or sustained [29]. Implementation knowledge should include the perspectives of key stakeholders, while also leveraging existing implementation science theory and evidence, to ensure implementation success.

To this end, we have selected several theoretically informed implementation measures, in addition to measuring the effectiveness of the Social Brain Toolkit. As social-ABI-lity and interact-ABI-lity are educational interventions, their effectiveness will be determined through measures of increased knowledge as well as ecologically valid postintervention measures such as frequency of social media use and confidence communicating with people with ABI. For the CPT intervention of convers-ABI-lity, effectiveness is determined by similarly meaningful measures of quality of life and conversation. The complexity of real-world implementation will be captured using mixed methods, including surveys and interviews exploring user satisfaction and experiences of implementation. For example, our specific survey of whether users are from rural, regional, remote, or metropolitan areas will enable the implementation experiences of these populations to be compared. Given the web-based nature of the Social Brain Toolkit, implementation measures will also include web analytics to identify any need for targeted implementation adjustments or strategies to improve intervention use. As the Social Brain Toolkit comprises technological interventions, think-aloud methods will be used to explore technological usability and provide users with a direct feedback channel to improve user interfaces.

As a prospective study of the real-world implementation of the Social Brain Toolkit, this study will not occur in a closed environment that allows controlled, randomized testing of isolated implementation strategies. Instead, with a theoretical foundation in the real-world complexity of digital health implementation [34], we will measure a comprehensive range of qualitative and quantitative effectiveness and implementation outcomes, and provide a recorded timeline of implementation strategy development and deployment for the Social Brain Toolkit.

### Strengths and Limitations

The strengths of this study include a hybrid implementation-effectiveness approach, a mixed methods design with a wide range of implementation measures collected from the outset of implementation, end-user–identified implementation strategies, and a strong theoretical underpinning in a digital health implementation framework. This study is constrained by some technical limitations regarding the collection of analytics, the sample size of the initial limited release of the Social Brain Toolkit interventions, and reliance on self-report for most outcome measures. However, these limitations will be acknowledged in reporting to assist appropriate interpretation.

### Conclusions

As people with ABI and their communication partners are the main intended beneficiaries of the Social Brain Toolkit, they have been and are being included from project inception to formative evaluation. Feedback provided by participants will directly inform future iterations of the interventions. Problems identified and recommendations made by users will be incorporated and addressed wherever possible in the next version of the Social Brain Toolkit. Therefore, beneficiaries will see concrete changes to interventions that directly reflect user input. These changes and their rationales will be documented and reported back to users in engaged scholarship that values and empowers stakeholder input through a direct feedback loop. The direct evaluation of the implementation of the interventions by end users in the community aims to ensure that the interventions are sufficiently feasible, acceptable, accessible, scalable, and sustainable to reach those in need of these supports in the community. The results can be used to improve the development and implementation of the Social Brain Toolkit, as well as future web-based psychosocial interventions for people with ABI and other populations.

## Appendix

Multimedia Appendix 1 Overview of prospective implementation data collection.

Multimedia Appendix 2 Overview of prospective effectiveness data collection.

Multimedia Appendix 3 convers-ABI-lity interview protocol for people with acquired brain injury and their communication partner.

Multimedia Appendix 4 convers-ABI-lity interview protocol for clinicians.

Multimedia Appendix 5 interact-ABI-lity interview protocol for informal communication partners.

Multimedia Appendix 6 interact-ABI-lity interview protocol for paid communication partners and clinicians.

Multimedia Appendix 7 social-ABI-lity interview protocol for people with acquired brain injury.

Multimedia Appendix 8 Entry surveys for people with acquired brain injury using convers-ABI-lity or social-ABI-lity.

Multimedia Appendix 9 Entry surveys for communication partners of people with acquired brain injury using convers-ABI-lity or interact-ABI-lity.

Multimedia Appendix 10 Exit surveys for all users of social-ABI-lity, convers-ABI-lity, and interact-ABI-lity.

Multimedia Appendix 11 Consenting process protocol, including relevant questions, adapted from the University of California, San Diego, Brief Assessment of Capacity to Consent instrument.

## Abbreviations

ABI: acquired brain injury
CPT: communication partner training
NASSS: Nonadoption, Abandonment, Scale-up, Spread, and Sustainability
UTS: University of Technology Sydney
