# Supplementary material for: A Web-Based Service Delivery Model for Communication Training After Brain Injury: Protocol for a Mixed Methods, Prospective, Hybrid Type 2 Implementation-Effectiveness Study
Source: JMIR Res Protoc. 2021 Dec 9;10(12):e31995. doi: 10.2196/31995 (PMC8704121; doi:10.2196/31995)
Supplement: Multimedia Appendix 1 [file resprot_v10i12e31995_app1.docx]

| Measure | | | | | | | Instrument or method | | Time points | |  |
| --- | --- | --- | --- | --- | --- | --- | --- | --- | --- | --- | --- |
| **Intervention: interact-ABI-lity** | | | | | | | | | | |  |
|  | Intervention reach | | | | | | User demographic information obtained through an entry survey specifically designed for this study | | Preintervention (primary time point). Cumulative participant responses will be collected over the 6-month implementation period | |  |
|  | User satisfaction and experience | | | | | | Exit survey specifically designed for this study | | Immediately after intervention (primary time point). Cumulative participant responses will be collected over the 6-month implementation period | |  |
|  | User satisfaction and experience | | | | | | Individual interviews with a minimum of 5 users of interact-ABI-lity | | As soon as possible after intervention (primary time point). Cumulative participant responses will be collected over the 6-month implementation period | |  |
|  | Adherence and fidelity | | | | | | Web analytics of (1) unique users; (2) modules completed; (3) quiz scores, answers submitted/recorded; (4) duration of course access; and (5) date/version of course accessed | | Six weeks after soft launch (ie, at 6 weeks), 6 weeks after full launch (ie, at approximately 4 months), and at 6 months (primary time point) | |  |
|  | Usability | | | | | | Survey specifically designed for this study | | Immediately after intervention (primary time point). Cumulative participant responses will be collected over the 6-month implementation period | |  |
|  | Usability | | | | | | Individual think-aloud interviews with a minimum of 5 users | | As soon as possible after intervention (primary time point). Cumulative participant responses will be collected over the 6-month implementation period | |  |
|  | User experience of implementation barriers and facilitators when completing the interventions | | | | | | Survey specifically designed for this study | | Immediately after intervention (primary time point). Cumulative participant responses will be collected over the 6-month implementation period | |  |
|  | User experience of implementation barriers and facilitators when completing the interventions | | | | | | Individual interviews (with a minimum of 5 users) of interact-ABI-lity | | As soon as possible after intervention (primary time point). Cumulative participant responses will be collected over the 6-month implementation period | |  |
|  | | | Web-based health care costs and equivalent face-to-face costs | | | | A bottom-up costing approach calculated using the following web analytics: (1) unique users; (2) modules completed; (3) quiz scores, answers submitted/recorded; and (4) duration of course access | | Postintervention, that is, at the end of the 6-month sampling frame of analytics (primary time point) | |  |
| **Intervention: social-ABI-lity** | | | | | | | | | | |  |
|  | | | Intervention reach | | | | | User demographic information obtained through an entry survey specifically designed for this study | | Preintervention (primary time point). Cumulative participant responses will be collected over the 6-month implementation period | |
|  | | | User satisfaction and experience | | | | | Survey specifically designed for this study | | Immediately after intervention (primary time point). Cumulative participant responses will be collected over the 6-month implementation period | |
|  | | | User satisfaction and experience | | | | | Individual interviews with a minimum of 5 users of social-ABI-lity (ie, people with ABI^a^) and any voluntary consenting participants who may have assisted the person to complete social-ABI-lity | | As soon as possible after intervention (primary time point). Cumulative participant responses will be collected over the 6-month implementation period | |
|  | | | Adherence and fidelity | | | | | Web analytics of (1) unique users; (2) modules completed; (3) quiz scores, answers submitted/recorded; (4) duration of course accessed; and (5) date/version of course accessed | | Six weeks after soft launch (ie, at 6 weeks), 6 weeks after full launch (ie, at approximately 4 months), and at 6 months (primary time point) | |
|  | | | Usability | | | | | Survey specifically designed for this study | | Immediately after intervention (primary time point). Cumulative participant responses will be collected over the 6-month implementation period | |
|  | | | Usability | | | | | Individual think-aloud interviews with a minimum of 5 users of social-ABI-lity (ie, people with ABI) and any voluntary consenting participants who may have assisted the person to complete social-ABI-lity | | As soon as possible after intervention (primary time point). Cumulative participant responses will be collected over the 6-month implementation period | |
|  | User experience of implementation barriers and facilitators when completing the interventions | | | | | | Survey specifically designed for this study | | Immediately after intervention (primary time point). Cumulative participant responses will be collected over the 6-month implementation period | |  |
|  | User experience of implementation barriers and facilitators when completing the interventions | | | | | | Individual interviews with a minimum of 5 users of social-ABI-lity (ie, people with ABI) and any voluntary consenting participants who may have assisted the person to complete social-ABI-lity | | As soon as possible after intervention (primary time point). Cumulative participant responses will be collected over the 6-month implementation period | |  |
|  | Web-based health care costs and equivalent face-to-face costs | | | | | | Bottom-up costing approach using the following web analytics: (1) unique users; (2) modules completed; (3) quiz scores, answers submitted/recorded; and (4) duration of course access | | Postintervention, that is, at the end of the 6-month sampling frame of analytics (primary time point) | |  |
| **Intervention: convers-ABI-lity** | | | | | | | | | | |  |
|  | | Intervention reach | | | | | User demographic information obtained through an entry survey specifically designed for this study | | Preintervention (primary time point). Cumulative participant responses will be collected over the 6-month implementation period | |  |
|  | | | | User satisfaction and experience | | | Survey specifically designed for this study for (1) 10 people with ABI and (2) their 10 familiar communication partners (eg, friends and family members) who have completed convers-ABI-lity, as well as (3) 5 clinicians delivering convers-ABI-lity | | Immediately after intervention (primary time point). Cumulative participant responses will be collected over the 6-month implementation period | |  |
|  | | | | User satisfaction and experience | | | Individual interviews with (1) 10 people with ABI and (2) their 10 familiar communication partners (eg, friends and family members) who have completed convers-ABI-lity, as well as (3) 5 clinicians delivering convers-ABI-lity | | As soon as possible after intervention (primary time point). Cumulative participant responses will be collected over the 6-month implementation period | |  |
|  | | | | Adherence and fidelity | | | Web analytics of (1) unique users; (2) bookings/cancellations and start/finish of telehealth appointments; and (3) date/version accessed | | Immediately after intervention for individual dyads (ie, person with ABI and their communication partner; primary time point). Cumulative participant responses will be collected over the 6-month implementation period | |  |
|  | | | | | Usability | | Survey specifically designed for this study with (1) 10 people with ABI and (2) their 10 familiar communication partners (e.g., friends and family members) who have completed convers-ABI-lity, as well as (3) 5 clinicians delivering convers-ABI-lity | | Immediately after intervention (primary time point). Cumulative participant responses will be collected over the 6-month implementation period | |  |
|  | | | | | Usability | | Individual think-aloud interviews with (1) 10 people with ABI and (2) their 10 familiar communication partners (e.g., friends and family members) who have completed convers-ABI-lity, as well as (3) 5 clinicians delivering convers-ABI-lity | | As soon as possible after intervention for individual dyads (ie, person with ABI and their communication partner; primary time point). Cumulative participant responses will be collected over the 6-month implementation period | |  |
|  | | | | | | User experience of implementation barriers and facilitators when completing the interventions | Survey specifically designed for this study, with (1) 10 people with ABI and (2) their 10 familiar communication partners (e.g., friends and family members) who have completed convers-ABI-lity, as well as (3) 5 clinicians delivering convers-ABI-lity | | Immediately after intervention for individual dyads (ie, person with ABI and their communication partner; primary time point). Cumulative participant responses will be collected over the 6-month implementation period | |  |
|  | | | | | | User experience of implementation barriers and facilitators when completing the interventions | Individual interviews with (1) 10 people with ABI and (2) their 10 familiar communication partners (eg, friends and family members) who have completed convers-ABI-lity, as well as (3) 5 clinicians delivering convers-ABI-lity | | As soon as possible after intervention (primary time point). Cumulative participant responses will be collected over the 6-month implementation period | |  |
|  | | | | | | Web-based health care costs and equivalent face-to-face costs | Bottom-up costing approach using web analytics of (1) unique users; (2) which pages are viewed and how often; and (3) bookings/cancellations and start/finish of telehealth appointments | | At the end of the 6-month sampling frame of web analytics (primary time point) | |  |

^a^ABI: acquired brain injury.
