# Supplementary material for: A Web-Based Service Delivery Model for Communication Training After Brain Injury: Protocol for a Mixed Methods, Prospective, Hybrid Type 2 Implementation-Effectiveness Study
Source: JMIR Res Protoc. 2021 Dec 9;10(12):e31995. doi: 10.2196/31995 (PMC8704121; doi:10.2196/31995)
Supplement: Multimedia Appendix 2 [file resprot_v10i12e31995_app2.docx]

| Intervention and measure | | Instrument or method | Time points |
| --- | --- | --- | --- |
| **interact-ABI-lity** | | | |
|  | Confidence interacting with people with ABI^a^ | Self-ratings in a questionnaire developed specifically for this study | Pretraining, immediately after training, and 3-month follow-up |
|  | Knowledge of appropriate communication strategies with people with ABI | Number of appropriate strategies generated by participants in each TBI^b^ case study; 2 experienced speech-language pathologists will review the lists of strategies generated by participants in each case study. The speech-language pathologists will code the strategies as appropriate or inappropriate using a consensus rating procedure | Pretraining, immediately after training, and 3-month follow-up |
|  | Knowledge of ABI, communication impairments, and effective communication with a person with ABI | Multiple-choice test developed specifically for this study | Pretraining and immediately after training |
| **social-ABI-lity** | | | |
|  | Confidence using social media | Self-ratings in a questionnaire developed specifically for this study | Pretraining, immediately after training, and 3-month follow-up |
|  | Knowledge of appropriate strategies for social media use | Number of appropriate strategies generated by participants in each TBI case study; 2 experienced speech-language pathologists will review the lists of strategies generated by participants in each case study. The speech-language pathologists will code the strategies as appropriate or inappropriate using a consensus rating procedure | Pretraining and immediately after training |
|  | Knowledge of effective and safe social media use | Multiple-choice test developed specifically for this study | Pretraining and immediately after training |
|  | Frequency of use of social media | Self-ratings in a questionnaire developed specifically for this study | Pretraining, immediately after training, and 3-month follow-up |
| **convers-ABI-lity** | | | |
|  | Perception of communicative ability | La Trobe Communication Questionnaire (self-report and other-report), completed on the web [3] | Initial assessment, postassessment (immediately after completion of final session), and 3-month follow-up |
|  | Conversation quality | Determined from conversation recordings using the TBI Conversation Rating Scales: Adapted Measure of Participation in Conversation and Measure of Support in Conversation [2] | Initial assessment, postassessment (immediately after completion of final session), and 3-month follow-up, as well as additional conversation practice tasks uploaded to convers-ABI-lity over the duration of the program |
|  | Conversation quality | Quantitative evaluation through facial expression (e.g., smile, frown, and engagement) measures on a scale of 0-100, generated from conversation recordings by custom iMotions software | Initial assessment, postassessment (immediately after completion of final session), and 3-month follow-up, as well as additional conversation practice tasks uploaded to convers-ABI-lity over the duration of the program |
|  | Quality of life | Quality of Life After Brain Injury Scale [3] | Initial assessment, postassessment (immediately after completion of final session), and 3-month follow-up |

^a^ABI: acquired brain injury.

^b^TBI: traumatic brain injury.

**References**

1. Douglas JM, O'Flaherty CA, Snow PC. Measuring perception of communicative ability: the development and evaluation of the La Trobe communication questionnaire. Aphasiology 2000 Mar; 14(3):251-68
2. Togher L, Power E, Tate R, McDonald S, Rietdijk R. Measuring the social interactions of people with traumatic brain injury and their communication partners: the adapted Kagan scales. Aphasiology 2010 Feb 03; 24(6-8):914-27
3. von Steinbüchel N, Wilson L, Gibbons H, Hawthorne G, Höfer S, Schmidt S, Bullinger M, Maas A, Neugebauer E, Powell J, von Wild K, Zitnay G, Bakx W, Christensen A, Koskinen S, Sarajuuri J, Formisano R, Sasse N, Truelle J, QOLIBRI Task Force. Quality of Life after Brain Injury (QOLIBRI): scale development and metric properties. J Neurotrauma 2010 Jul; 27(7):1167-85
