# Supplementary material for: A Web-Based Service Delivery Model for Communication Training After Brain Injury: Protocol for a Mixed Methods, Prospective, Hybrid Type 2 Implementation-Effectiveness Study
Source: JMIR Res Protoc. 2021 Dec 9;10(12):e31995. doi: 10.2196/31995 (PMC8704121; doi:10.2196/31995)
Supplement: Multimedia Appendix 3 [file resprot_v10i12e31995_app3.docx]

| Domain | Question for people with acquired brain injury (ABI) and their communication partner (CP) | Follow up questions |
| --- | --- | --- |
| 1. FOUR: Adopters | - Can you (think-aloud screenshare - Workflow or equivalent) show me and talk me through the exact order you used the portal for a session? - Which steps of that process took some getting used to? - Which steps were easy/straightforward? - How did you find the process of using GAS (Goal attainment scales)? - What would you do differently next time? | - Why? - Could you give me an example? |
| 1. ONE: Condition | - What made it easy/ hard to use convers-ABI-lity with a brain injury/ as a communication partner? |  |
| 1. TWO: Technology | - What was it like using the computer/internet to   1. Make recordings?   2. Make appointments?   3. Upload recordings?   4. Watch videos?   5. Answer questions?   6. Type answers? |  |
| 1. SEVEN: Over time | - What would you improve/change about convers-ABI-lity? |  |
| 1. THREE: Value proposition | - What made you want to try convers-ABI-lity? - As someone with/who talks a lot to someone with a brain injury/stroke, what did you get out of convers-ABI-lity? - What was the most significant change for you as a result of completing the training? - What impact has this change had on your life? - On a scale of 1 to 10, how likely would you be to recommend this training to others? - If we make the course available to speech-language pathologists and families in the future, who do you think should cover the costs? (e.g., families, speech-language pathologists, insurers like icare, government schemes like the NDIS or Medicare?) - What would be a fair price for [insert participant’s answer from previous question] to pay for the course? - Probe charging (service, access codes for individual clients, assessment/manual costs that much, how do you think) |  |
| 1. SIX: Wider System | - How do you feel about   1. using online therapy?   2. having your progress recorded online?   3. paying for convers-ABI-lity or it being covered by Medicare/insurer/NDIS? |  |
