# Supplementary material for: A Web-Based Service Delivery Model for Communication Training After Brain Injury: Protocol for a Mixed Methods, Prospective, Hybrid Type 2 Implementation-Effectiveness Study
Source: JMIR Res Protoc. 2021 Dec 9;10(12):e31995. doi: 10.2196/31995 (PMC8704121; doi:10.2196/31995)
Supplement: Multimedia Appendix 4 [file resprot_v10i12e31995_app4.docx]

| Domain | Question for clinician | Follow up questions |
| --- | --- | --- |
| 1. FOUR: Adopters | - Can you (think-aloud screenshare - Workflow or equivalent) show me and talk me through the exact order you used the portal for a session? - Which steps of that process took some getting used to? - Which steps were easy/straightforward? - How did you find the process of using GAS (Goal attainment scales)? - What would you do differently next time? | - Why? - Could you give me an example? |
| 1. ONE: Condition | - What adjustments did you make to convers-ABI-lity because of your clients’ brain injury? |  |
| 1. TWO: Technology | - What was it like using the computer/internet to   1. Run a session?   2. Make appointments?   3. Record notes?   4. Review progress? |  |
| FIVE: Organization | - What did [name of practice/service] have to put in place to start using convers-ABI-lity? - How easy or difficult was this to do? |  |
| SEVEN: Over time | - What would you improve/ do differently next time? - Over time, what would [name of practice/service] have to do differently to keep offering convers-ABI-lity? |  |
| THREE: Value proposition | - What made you want to try convers-ABI-lity? - What did you get out of convers-ABI-lity as a clinician and service? - What was the most significant change for you as a result of delivering the training? - What impact has this change had on your practice? - On a scale of 1 to 10, how likely would you be to recommend this training to others? - If we make the course available to speech-language pathologists and families in the future, who do you think should cover the costs? (e.g., families, speech-language pathologists, insurers like icare, government schemes like the NDIS or Medicare?) - What would be a fair price for [insert participant’s answer from previous question] to pay for the course? - Probe charging (service, access codes for individual clients, assessment/manual costs that much, how do you think) |  |
| SIX: Wider System | - How do you feel about   1. Delivering online therapy?   2. Collecting and recording progress online?   3. paying for convers-ABI-lity or it being covered by Medicare/insurer/NDIS? |  |
