# Supplementary material for: A Web-Based Service Delivery Model for Communication Training After Brain Injury: Protocol for a Mixed Methods, Prospective, Hybrid Type 2 Implementation-Effectiveness Study
Source: JMIR Res Protoc. 2021 Dec 9;10(12):e31995. doi: 10.2196/31995 (PMC8704121; doi:10.2196/31995)
Supplement: Multimedia Appendix 5 [file resprot_v10i12e31995_app5.docx]

| Domain | Question for informal communication partner | Follow up questions |
| --- | --- | --- |
| FOUR: Adopters | - Can you (think-aloud screenshare - Workflow or equivalent) show me and talk me through the exact order you used the course? - Which steps of that process took some getting used to? - Which steps were easy/straightforward? | - Why? - Could you give me an example? |
| ONE: Condition | - What part of the course was/not relevant to you as a communication partner of someone with [condition]? |  |
| TWO: Technology | - What was it like using the computer/internet to   1. Watch videos?   2. Answer questions?   3. Type answers? |  |
| SEVEN: Over time | - What would you improve/change about the course? |  |
| THREE: Value proposition | - What made you want to try the course? - As someone with/who talks a lot to someone with a brain injury/stroke, what did you get out of the course? - What was the most significant change for you as a result of completing the training? - What impact has this change had on your life? - On a scale of 1 to 10, how likely would you be to recommend this training to others? - If we make the course available to speech-language pathologists and families in the future, who do you think should cover the costs? (e.g., families, speech-language pathologists, insurers like icare, government schemes like the NDIS or Medicare?) - What would be a fair price for [insert participant’s answer from previous question] to pay for the course? |  |
| SIX: Wider System | - How do you feel about   1. Learning online?   2. Having your progress recorded online? |  |
