# Supplementary material for: A Web-Based Service Delivery Model for Communication Training After Brain Injury: Protocol for a Mixed Methods, Prospective, Hybrid Type 2 Implementation-Effectiveness Study
Source: JMIR Res Protoc. 2021 Dec 9;10(12):e31995. doi: 10.2196/31995 (PMC8704121; doi:10.2196/31995)
Supplement: Multimedia Appendix 9 [file resprot_v10i12e31995_app9.docx]

| Domain | Question for communication partner | Question Type |
| --- | --- | --- |
| Adopters | - Age (range)   1. Under 18   2. 18-24   3. 25-34   4. 34-45   5. 45-54   6. 55-64   7. 64+ - Gender   1. Male   2. Female   3. Other   4. Prefer not to say - How frequently do you use the internet?   1. Several times a day   2. Daily   3. Several times a week   4. Weekly   5. Monthly   6. Rarely - What is the highest education level you have completed?   1. Currently a school student   2. Year 6 equivalent / age 12 (e.g., Primary school)   3. Year 10 equivalent / age 16 (e.g., School certificate)   4. Year 12 equivalent / age 18 (e.g., Higher school certificate)   5. Certificate/Diploma after high school   6. Bachelor's Degree (Undergraduate)   7. Graduate Diploma/Certificate (Postgraduate)   8. Master's Degree (Postgraduate degree)   9. PhD - Was English your first language? (Yes/No) - How would you describe your location? Metro/ Regional/Rural/Remote - What is your relationship to a person with communication difficulty?   1. Paid support worker   2. Partner   3. Family   4. Friend - How long have you been their communication partner after their injury/stroke/condition?   1. Less than 6 months   2. 6-12 months   3. More than 12 months | Multiple choice |
| Organization | - How did you hear about this course?   1. Therapist   2. Social media   3. Friend/Family   4. Internet search   5. Research conference   6. Professional groups   7. Other (please specify) | Multiple choice |
| Wider System | - Country | Short answer/ Drop down |
