# Supplementary material for: A Web-Based Service Delivery Model for Communication Training After Brain Injury: Protocol for a Mixed Methods, Prospective, Hybrid Type 2 Implementation-Effectiveness Study
Source: JMIR Res Protoc. 2021 Dec 9;10(12):e31995. doi: 10.2196/31995 (PMC8704121; doi:10.2196/31995)
Supplement: Multimedia Appendix 10 [file resprot_v10i12e31995_app10.docx]

| Domain | Question | Question Type |
| --- | --- | --- |
| Overall | - How easy was this course to complete?   1. very easy   2. easy,   3. somewhat easy   4. hard,   5. Very hard, | 5 point Scale |
|  | - Why do you think so? | Paragraph |
| Condition | - As a person with brain injury/stroke/other condition, how much did your symptoms (e.g. difficulty with memory, attention/concentration, planning or communication) affect your experience of this course? - As a communication partner of someone with a brain injury/stroke/other condition, how much do you think symptoms of brain injury/stroke (e.g. difficulty with memory, attention/concentration, planning or communication) affected their experience of this course? | 5 point Scale |
| Technology | - What was it like to do the course online on the computer/device?   1. very easy   2. easy,   3. neither easy nor hard   4. Very hard, | 5 point Scale |
|  | - Did you experience technological difficulty during this course?   1. A lot   2. Quite a lot   3. Some   4. A little   5. None | 5 point Scale |
| Value proposition | - My knowledge of communication and brain injury improved doing this course   1. Strongly agree   2. Agree   3. Neutral   4. Disagree   5. Strongly agree - What was the most useful part of the course? (short answer) | 5 point Scale |
| Adopters | - Was there anything that made this course hard to finish? - What helped you finish the course? | Short answers |
| Over time | - What would you improve/change about this course? | Paragraph |
