# Supplementary material for: A Web-Based Service Delivery Model for Communication Training After Brain Injury: Protocol for a Mixed Methods, Prospective, Hybrid Type 2 Implementation-Effectiveness Study
Source: JMIR Res Protoc. 2021 Dec 9;10(12):e31995. doi: 10.2196/31995 (PMC8704121; doi:10.2196/31995)
Supplement: Multimedia Appendix 11 [file resprot_v10i12e31995_app11.pdf]

Protocol for Consent Assessment / Support Process

Capacity for Consent (Q 1-5) – questions adapted from University of California, San Diego Brief Assessment of Capacity to Consent (UBACC) (Jeste et al., 2007)

Question	Required Answer
**1. Do you believe this is primarily research or primarily treatment?**	Research
**2. Do you have to be in this study if you do not want to participate?**	No
**3. If you withdraw from the study, will you still be able to receive regular treatment?**	Yes
**4. If you participate in this study, what are some of the things you will be asked to do?**	Video Call/ interview
**5. Please describe some of the risks or discomforts people may experience if they participate in this study.**	Any synonyms for psychological distress (e.g. Tired/ Fatigue, upset)

- A speech pathologist researcher from the research team will be available via video call at an agreed time to support people with ABI to complete and sign the electronic consent form

- Potential participants will be allowed to refer to the participant information statement as a support when responding to the questions.

- If the potential participant responds incorrectly to a question, the researcher will provide further explanation about the study, and then verify comprehension of the information.

- If the potential participant is unable to reliably demonstrate comprehension of this key information about the study, the participant will be excluded from participation.

Verbal Consent (Q 6 onwards) – questions adapted from UTS Verbal Consent template and UTS Consent Form Template

Consent	Answers	
**6. Have you read the information contained in the participant information sheet?**	Yes	No
**7. Have you had an opportunity to ask questions and are you satisfied with the answers you have received?**	Yes	No

**8. Do you understand the purposes, procedures and risks of the research described in the Participant Information Sheet?**	Yes	No
**9. Do you understand that the research will produce reports, academic work, articles, conference presentations?**	Yes	No
**10. Do you freely agree to participate in this activity, with the understanding that you may withdraw at any time?**	Yes	No
**11. Do you agree to be audio and video recorded during the video forum?**	Yes	No
**12. Do you agree to have your survey answers recorded?**	Yes	No
**13. Do you agree that the research data gathered from this project may be published and presented in a form that does not identify you in any way**	Yes	No
**14. Do you agree that the research data gathered from this project may be used for future research purposes?**	Yes	No
**15. Are you aware that you can contact Melissa Miao at melissa.miao@uts.edu.au at any time and/or to arrange a call in your time zone if I have any concerns about the research?**	Yes	No
**16. Which speech pathologist helped you to complete this form?**	Melissa Emma Rachael Liss	

- If potential participants answered NO to any of these – clarify, verify understanding and/or discontinue interview as appropriate
